# Pretreatment plasma fibrinogen level as a prognostic biomarker for patients with lung cancer

**DOI:** 10.6061/clinics/2020/e993

**Published:** 2020-02-18

**Authors:** Yi Zhang, Junyan Cao, Yinan Deng, Yiming Huang, Rong Li, Guozhen Lin, Min Dong, Zenan Huang

**Affiliations:** I Department of Hepatic Surgery, The Third Affiliated Hospital of Sun Yat-sen University, Guangzhou, Guangdong, China; II Department of Medical Ultrasound, The Third Affiliated Hospital of Sun Yat-sen University, Guangzhou, Guangdong, China; III Guangdong Key Laboratory of Liver Disease Research, The Third Affiliated Hospital of Sun Yat-sen University, Guangzhou, Guangdong, China; IV Department of Medical Oncology, The Third Affiliated Hospital of Sun Yat-sen University, 600 Tianhe Road, Tianhe District, Guangzhou 510000, Guangdong, China.; V Breast Cancer Center, The Third Affiliated Hospital of Sun Yat-sen University, 600 Tianhe Road, Tianhe District, Guangzhou 510000, Guangdong, China

**Keywords:** Pretreatment Plasma Fibrinogen, Lung Cancer, Prognosis, Meta-Analysis

## Abstract

Many researchers have shown that pretreatment plasma fibrinogen levels are closely correlated with the prognosis of patients with lung cancer (LC). In this study, we thus performed a meta-analysis to systematically assess the prognostic value of pretreatment plasma fibrinogen levels in LC patients.

A computerized systematic search in PubMed, EMBASE, Web of Science and China National Knowledge Infrastructure (CNKI) was performed up to March 15, 2018. Studies with available data on the prognostic value of plasma fibrinogen in LC patients were eligible for inclusion. The pooled hazard ratios (HRs) and odd ratios (ORs) with 95% confidence intervals (CIs) were used to evaluate the correlation between pretreatment plasma fibrinogen levels and prognosis as well as clinicopathological characteristics.

A total of 17 studies with 6,460 LC patients were included in this meta-analysis. A higher pretreatment plasma fibrinogen level was significantly associated with worse overall survival (OS) (HR: 1.57; 95% CI: 1.39-1.77; *p*=0.001), disease-free survival (DFS) (HR: 1.53; 95% CI: 1.33-1.76; *p*=0.003), and progression-free survival (PFS) (HR: 3.14; 95% CI: 2.15-4.59; *p*<0.001). Furthermore, our subgroup and sensitivity analyses demonstrated that the pooled HR for OS was robust and reliable. In addition, we also found that a higher fibrinogen level predicted advanced TNM stage (III-IV) (OR=2.18, 95% CI: 1.79-2.66; *p*<0.001) and a higher incidence of lymph node metastasis (OR=1.74, 95% CI: 1.44-2.10; *p*=0.02).

Our study suggested that higher pretreatment plasma fibrinogen levels predict worse prognoses in LC patients.

## INTRODUCTION

Lung cancer (LC) remains a major cancer-associated cause of death worldwide. LC falls into either non-small cell lung cancer (NSCLC) or small cell lung cancer (SCLC), with NSCLC accounting for nearly 85% of incidents (1,2). Although there have been great advances in LC therapies, including surgery, chemoradiotherapy and molecular targeted drugs during the past decade, LC patients still have rather low 5-year survival rates (<18%) (2). Disease recurrence and distant metastasis play a critical role in deteriorating the prognosis of LC patients (3-5). To date, several clinicopathological characteristics have been used to predict recurrence, distant metastasis and oncological survival in LC patients, such as differentiation grade, tumor location and TNM stage (6). The TNM staging system established based on the Union for International Cancer Control (UICC) and the International Association for the Study of Lung Cancer (IASLC) TNM classification is of great importance and usefulness in predicting oncological prognoses and guiding individual treatment, but the overall survival (OS) of NSCLC patients varies widely, even for patients with the same TNM stage (6). Therefore, identifying novel reliable biomarkers for obtaining additional prognostic information on LC is essential, especially robust, economical, and easily accessible biomarkers.

An increasing amount of evidence has shown that there is a close association between the activation of coagulation and fibrinolysis and cancer invasion, angiogenesis and metastasis (7-13). Additionally, numerous clinical studies suggested that hemostatic abnormalities and many hemostasis markers closely correlated with tumor stage and the oncological long-term outcomes of cancer patients (14). Fibrinogen, as a molecule produced by the liver in response to cytokine stimulation, is a key element of the coagulation system. Many studies have reported that the pretreatment plasma fibrinogen level was significantly associated with the tumor pathological stage and response to therapy and could act as a negative predictor of prognosis in LC patients (2,6,14-26). Nevertheless, the weight of its prognostic value in LC is still debated since many of the studies investigating the prognostic value of fibrinogen enrolled only small sample sizes, suggesting that their conclusions may have low statistical power. Therefore, in this study, we performed a systematic review and meta-analysis of the previously published literature to comprehensively assess the prognostic value of pretreatment plasma fibrinogen levels in LC patients.

## MATERIALS AND METHODS

### Study selection

We systemically searched the accessible databases, including PubMed, Web of Science, EMBASE and China National Knowledge Infrastructure (CNKI), to identify eligible studies published up to March 15, 2018. Three key search items were used during this process: (fibrinogen or hyperfibrinogenemia), (lung cancer/carcinoma or pulmonary cancer/carcinoma) and (prognosis or prognostic or survival or outcome). The search strategy used in PubMed was as follows: search ((fibrinogen[Title/Abstract]) OR hyperfibrinogenemia[Title/Abstract]) AND ((((lung cancer[Title/Abstract]) OR lung carcinoma[Title/Abstract]) OR pulmonary cancer[Title/Abstract]) OR pulmonary carcinoma [Title/Abstract]) AND ((((prognosis[Title/Abstract]) OR prognostic[Title/Abstract]) OR survival[Title/Abstract]) OR outcome[Title/Abstract]). Additionally, we manually checked the reference lists in relevant reviews and retrieved studies to identify additional potentially eligible studies.

### Inclusion and exclusion criteria

The inclusion criteria were as follows: (1) clinical study focusing on exploring the prognostic value of pretreatment plasma fibrinogen in LC. (2) Hazard ratios (HRs) with 95% confidence intervals (CIs) for survival outcomes were presented directly, including OS and/or disease-free survival (DFS) and progression-free survival (PFS), or these values could be obtained by analyzing the Kaplan-Meier (KM) curve provided in the eligible studies. Otherwise, the publications were excluded if they were letters, reviews, duplicates, studies without available HRs for OS, DFS or PFS, and studies not involving the association between pretreatment fibrinogen and prognosis in LC.

### Data collection and quality assessment

Two authors extracted the relevant data in an independent manner. The extracted data included the first author, publication year, country, recruitment period, median age, study sample size, histological type of LC, tumor TNM stage, cut-off values for high fibrinogen level, treatment type, follow-up time, HRs with 95% CIs for survival outcomes, and analysis type. During this process, any discrepancies were resolved by discussion among all the authors.

In this meta-analysis, we applied the Newcastle–Ottawa Scale (NOS) score to evaluate the quality of each included study (27). The NOS criteria contain eight items that are categorized into three domains, including selection, comparability, and exposure. A maximum of one star is awarded to each item with high quality in selection and exposure, while the item related to comparability can be given a maximum of two stars. The NOS score ranges from zero to nine stars, and studies with at least 6 stars were considered to have high quality.

### Statistical analysis

The combined HRs and their corresponding 95% CIs were calculated to assess the relationship between pretreatment plasma fibrinogen and survival outcomes. The combined odds ratios (ORs) and their corresponding 95% CIs were used to quantitatively determine the link between plasma fibrinogen and the clinicopathological parameters of patients with LC. The coexistence of HR or OR>1 (low fibrinogen as reference), 95% CI not overlapping 1, and *p-*value <0.05 indicated worse OS and more unfavorable clinicopathological parameters. We assessed the statistical heterogeneity across the studies using Cochran’s *Q* test and Higgins *I* ^2^ statistic. A random-effects model was used to calculate the parameters when there was significant heterogeneity across studies (*I* ^2^>50% indicated high heterogeneity). Otherwise, a fixed-effects model was applied. Subgroup analysis, stratified by country, sample size, TNM stage, cut-off value, treatment type, starting time of recruitment period and analysis type, was performed to investigate the potential sources of heterogeneity and to concurrently test the reliability of the total pooled results. In addition, we performed sensitivity analysis to further test the robustness of the total pooled results by the sequential omission of individual studies. Publication bias was evaluated using Begg’s funnel plots (28) and Egger’s test (29). If one of the two tests suggested that obvious publication bias existed among the included studies, the trim-and-fill method was applied to determine whether the publication bias significantly affected the robustness and reliability of the pooled results. All statistical manipulations in this meta-analysis were carried out using Stata 12.0 software (Stata Corporation, College Station, TX), and a *p*-value >0.05 was used as the threshold of statistical significance.

## RESULTS

### Study identification

**Figure 1:**
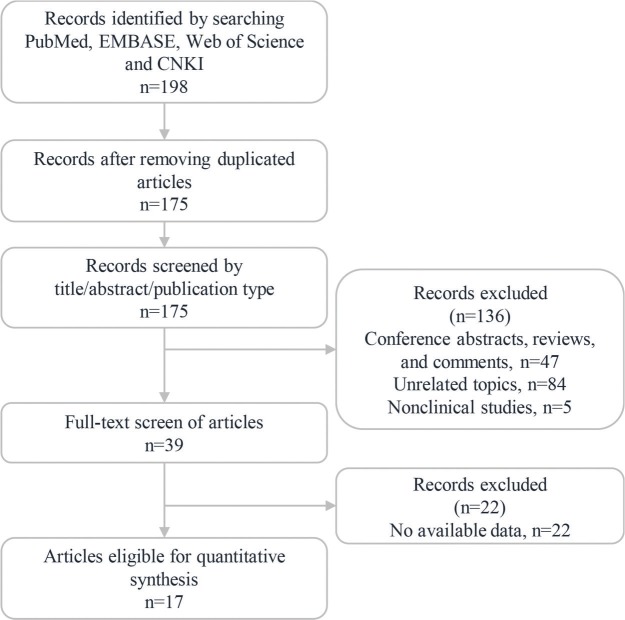
Flow diagram of the literature identification process.

The study identification process is summarized in Figure 1. A total of 198 articles were retrieved from the PubMed, EMBASE, Web of Science and CNKI databases in the initial search. Among them, a total of 23 duplicated articles were excluded using EndNote X7 software. The remaining 175 publications were further assessed by checking the titles and abstracts, and 136 articles were excluded because they were reviews, comments, conference abstracts, cell/animal experiments or had irrelevant topics. Subsequently, a total of 39 studies were evaluated by screening their full texts, and 22 studies were excluded since they did not provide sufficient data. Ultimately, 17 eligible studies were included in this meta-analysis (2,6,14-26,30,31).

### Study characteristics and quality

Our meta-analysis included a total of 6,460 patients from China (n=15) (2,6,12,14,16-23,25,26,31), Turkey (n=1) (15), and Korea (n=1) (30). Of all the included studies, 2 studies enrolled patients with SCLC (14,31), 14 studies enrolled patients with NSCLC (2,6,16-26,30) and 1 study enrolled a mixed population (15). There were inconsistencies in the cut-off values of the high plasma fibrinogen level among the included studies. More information on the main characteristics of the 17 included studies is summarized in Table 1. With respect to the quality assessment, all of the included studies had scores ranging from 6 to 8, indicating that they were of high quality (Table 2).

### Meta-analysis

#### Association between fibrinogen level and prognosis in LC

**Figure 2:**
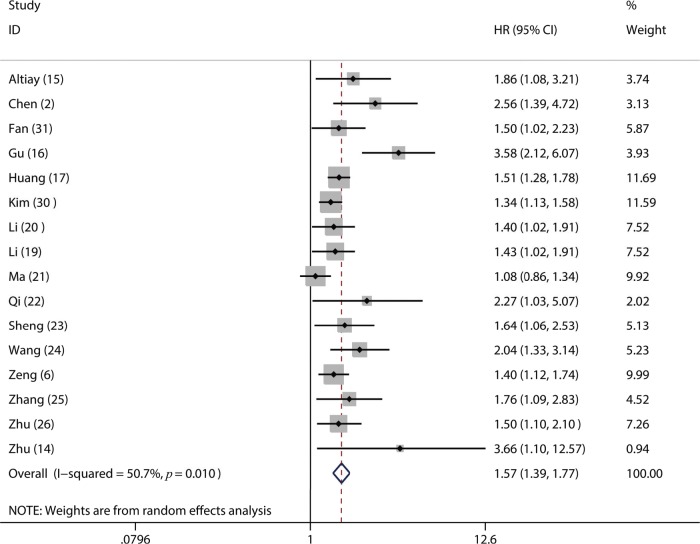
Forest plot of the hazard ratio (HR) for the association between the plasma fibrinogen level and overall survival (OS) in patients with lung cancer.

**Figure 3:**
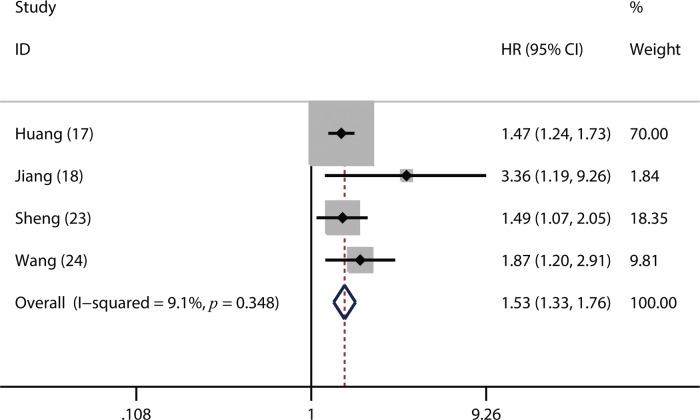
Forest plot of the hazard ratio (HR) for the association between the plasma fibrinogen level and disease-free survival (DFS) in patients with lung cancer.

**Figure 4:**
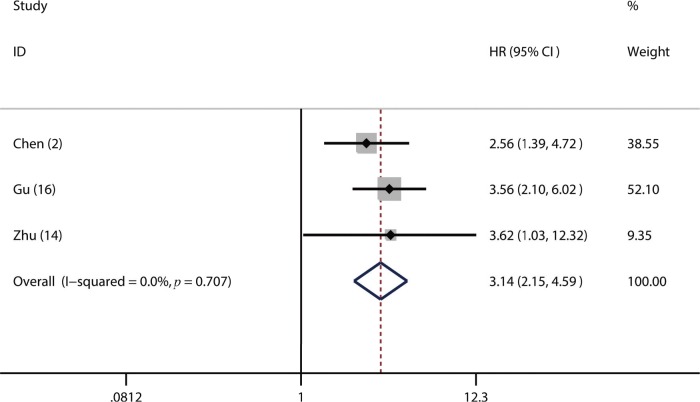
Forest plot of the hazard ratio (HR) for the association between the plasma fibrinogen level and progression-free survival (PFS) in patients with lung cancer.

Of the included studies, 16 studies reported OS (2,6,14-17,19-26,30,31), four studies referred to DFS (17,18,23,24) and 3 studies involved PFS (2,14,16). From the pooled results, we found that a higher pretreatment plasma fibrinogen level was significantly associated with worse OS (HR: 1.57; 95% CI: 1.39-1.77; *p*=0.001; Figure 2), DFS (HR: 1.53; 95% CI: 1.33-1.76; *p*=0.003; Figure 3), and PFS (HR: 3.14; 95% CI: 2.15-4.59; *p*<0.001; Figure 4).

#### Association between fibrinogen level and clinicopathological parameters in LC

**Figure 5:**
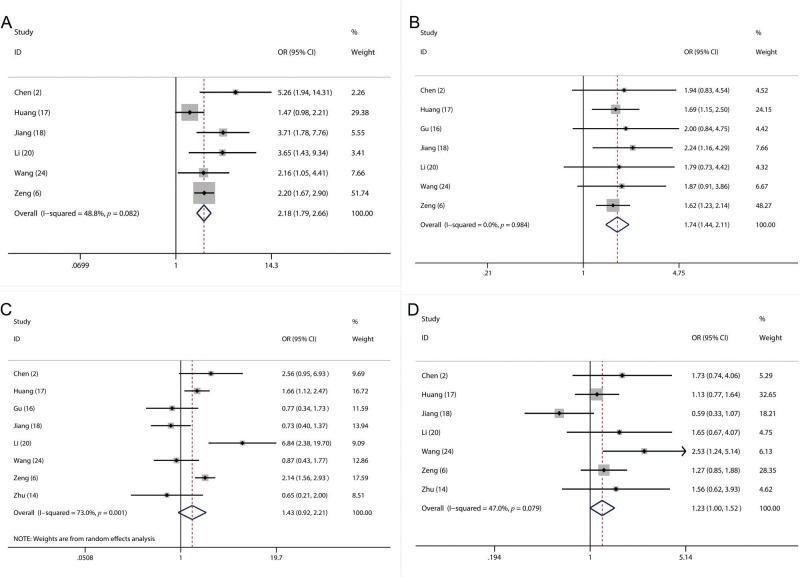
Forest plot of the odds ratios (ORs) for the association of the plasma fibrinogen level with TNM stage (A), lymph node metastasis (B), sex (C) and age (D).

In this meta-analysis, sufficient data were available for analyzing the association between the fibrinogen level and several clinicopathological parameters, including sex (2,6,14,16-18,20,24), age (2,6,14,17,18,20,24), lymph node metastasis (2,6,16-18,20,24) and TNM stage (2,6,17,18,20,24). We found that a higher fibrinogen level was significantly related to more advanced TNM stage (III-IV) (OR=2.18, 95% CI: 1.79-2.66; *p*<0.001; Figure 5A) and a higher frequency of lymph node metastasis (OR=1.74, 95% CI: 1.44-2.10; *p*=0.02; Figure 5B). However, our results showed that there were no significant associations between the plasma fibrinogen level and sex (OR=1.43, 95% CI: 0.92-2.21; *p*=0.08; Figure 5C) or age (OR=1.23, 95% CI: 1.00-1.52; *p*=0.11; Figure 5D).

### Subgroup and sensitivity analyses

**Figure 6:**
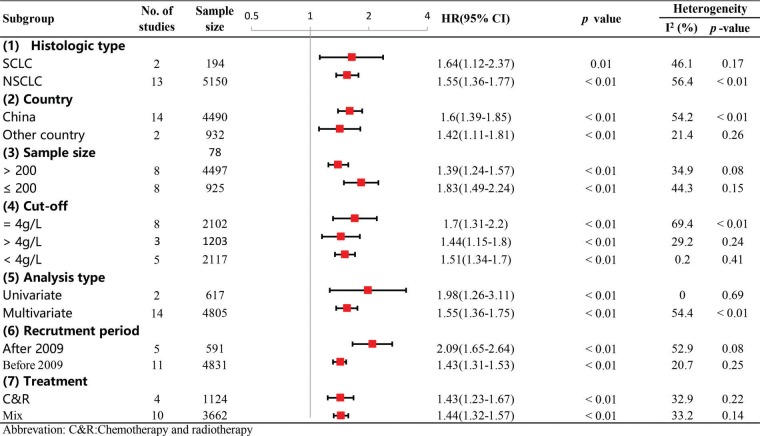
Subgroup analysis of exploring the possible sources of heterogeneity for the pooled HR for OS.

**Figure 7:**
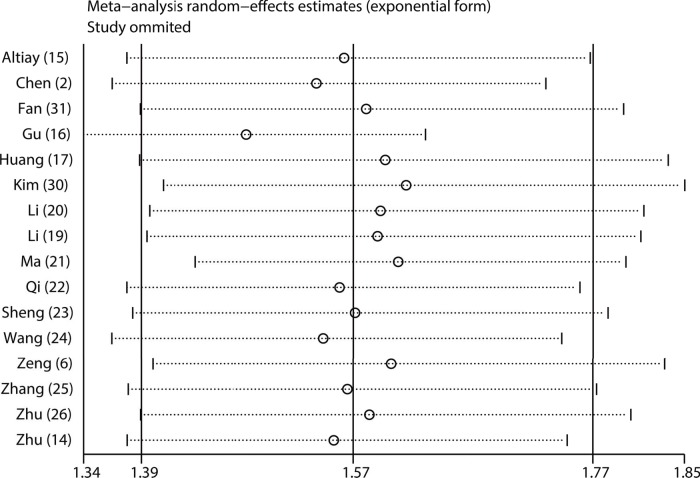
Sensitivity analyses of assessing the robustness of the pooled HR for OS.

To investigate the possible sources of heterogeneity for the pooled HR of OS, subgroup analysis was performed according to country, sample size, recruitment time, cut-off value, treatment type, histological type and analysis type. From the results, we observed that significant heterogeneity did not continuously exist in the subgroups of sample size, recruitment period and treatment type (Figure 6), suggesting that they might partly explain the sources of heterogeneity in our meta-analysis. In addition, the results of all the subgroup analyses showed that the pooled HRs for OS were still more than 1, and their CIs did not overlap 1 (Figure 6), indicating that our pooled result regarding OS was robust and reliable. Moreover, our sensitivity analysis showed that the pooled HRs for OS were not altered significantly when individual studies were omitted in each step, which further demonstrated the stability and dependability of our pooled result regarding OS (Figure 7).

### Evaluation of publication bias

**Figure 8:**
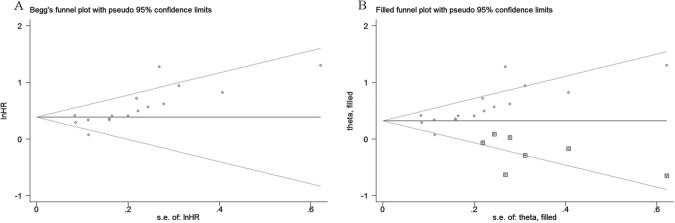
Begg’s funnel plot of evaluating the publication bias for OS (A). Adjusted Begg’s funnel plot from the trim-and-fill method for assessing the publication bias for OS (B).

Begg’s test and Egger’s test were used to evaluate publication bias for OS. Our results showed that Begg’s funnel plot appeared asymmetric (Figure 8A), and a *p-*value <0.05 was obtained in Egger’s test, indicating that significant publication bias existed among these studies exploring the association between the fibrinogen level and OS in LC. To evaluate the degree of the influence that the publication bias had on the dependability of the pooled HR for OS, the trim-and-fill method was used in this analysis. The results showed that the adjusted funnel plot became symmetric (Figure 8B). More importantly, from the results, we found that the adjusted pooled HR for OS was still more than 1, and its corresponding CI did not overlap 1. Thus, the results of the trim-and-fill method verified that the publication bias did not significantly affect the reliability of the pooled result regarding the link between higher fibrinogen levels and worse OS. Because there were less than 10 eligible studies that reported DFS/RFS, PFS and clinicopathological parameters, it was statistically insignificant to perform Begg’s test and Egger’s test for assessing publication bias among the eligible studies.

## DISCUSSION

The association between pretreatment plasma fibrinogen levels and prognosis has been investigated in a variety of malignant tumors, including LC. However, no meta-analyses have been conducted to systematically assess the correlation between the plasma fibrinogen level and prognosis as well as the clinicopathological characteristics of LC patients. To the best of our knowledge, this study is the first meta-analysis to systematically evaluate the prognostic value of pretreatment plasma fibrinogen levels in LC.

In this meta-analysis, a total of 17 eligible studies were finally included, of which 14 focused on NSCLC, 2 focused on SCLC and 1 focused on the mixed populations of NSCLC and SCLC. We found that a higher fibrinogen level was significantly associated with worse OS, DFS and PFS in LC patients. Furthermore, our subgroup analysis and sensitivity analysis verified the robustness and reliability of our pooled HR for OS. In particular, the multivariate analysis of the subgroups showed that the pooled HR for OS continued to be over 1, with its CI not overlapping 1, which indicated that the pretreatment plasma fibrinogen level may be an independent prognostic factor for LC patients. In addition, we also found that a higher fibrinogen level was significantly related to advanced TNM stage and a higher frequency of lymph node metastasis.

Several biological mechanisms may explain the prognostic significance of higher pretreatment plasma fibrinogen levels in LC patients. First, fibrinogen is a biomarker that could reflect the status of the tumor-associated inflammatory response (32). Many tumor growth and metastasis-enhancing events always occur during the tumor-associated inflammatory response, such as the remodeling of the extracellular matrix, the induction of angiogenesis, the inhibition of apoptosis, the enactment of immunosuppressive effects, the stimulation of DNA damage and the increased release of cytokines and inflammatory mediators (33). For instance, an animal experiment showed that clearing the fibrinogen in circulation could inhibit the formation of pulmonary metastases (9), at least suggesting that fibrinogen could reflect a status availing tumor progression. Second, fibrinogen could bind to some growth factors, such as vascular endothelial growth factor and fibroblast growth factor, and facilitate these growth factors binding to their receptors on the tumor cell surface, which plays a crucial role in contributing to tumor proliferation and angiogenesis (34). Third, fibrinogen deposition could promote thrombosis by strengthening the interaction of cancer cells and platelets, assisting tumor cells in escaping from the killing effects of natural killers (12). Overall, these mechanical studies largely increased the reliability of our findings in this meta-analysis.

Apart from the interesting findings, our analysis has several limitations as well. First, studies published in only English and Chinese were included, and studies published in other languages were not considered, which may introduce publication bias. Second, the estimated HR was abstracted from the KM survival curve due to a lack of original data, likely causing some statistical errors and affecting the precision of the estimation. Third, the included studies had inconsistent TNM stages and different accrual periods, therefore leading to some heterogeneity in our meta-analysis. Fourth, the majority of the included studies were conducted in China, so more clinical studies should be performed to assess the association between plasma fibrinogen and the prognosis of LC patients from other regions. There may be three potential reasons why most of the included studies were performed in China. First, LC is the leading cause of cancer-related death, and its incidence and mortality in China have substantially increased in recent years as a consequence of lifestyle diversity and socioeconomic development (35). In particular, lung cancer-associated mortality in China is higher than that in most countries (35). Thus, it is likely that more Chinese researchers focused on exploring novel reliable biomarkers for predicting prognosis and optimizing individualized therapy for patients with LC. Second, we performed the literature selection in this meta-analysis with language restrictions of English and Chinese since we are not fluent in other languages. Thus, there is a possibility that the language restrictions in this meta-analysis probably excluded some eligible studies published in other languages and subsequently increased the risk of bias, which may also partly account for why most of the included studies were performed in China. Third, in this meta-analysis, the publication bias assessment suggested that there was significant publication bias, and the trim-and-fill analysis indicated that 7 studies may not be permitted for publication owing to the negative results on the prognostic value of the pretreatment plasma fibrinogen level in patients with LC. It may be coincidental that the 7 studies with negative results were performed in other countries, which may partly explain why only 2 studies from other countries were included as well. Fifth, the cut-off values for high fibrinogen levels differed across the included studies, ranging from 3.3 and 5.8, which probably also introduced heterogeneity into our meta-analysis. Further studies are needed to determine an optimal cut-off value that provides more precise guidance for individual treatment. Sixth, only two eligible studies with small sample sizes provided available data regarding the association between the fibrinogen level and prognosis in SCLC. Hence, more high-quality studies with larger sample sizes are needed to further investigate the association between the fibrinogen level and prognosis in SCLC.

In conclusion, our study showed that high pretreatment plasma fibrinogen levels predicted a worse prognosis in LC. However, more prospectively well-designed studies are needed to further confirm this conclusion due to some aforementioned limitations.

## AUTHOR CONTRIBUTIONS

Huang Z and Zhang Y conceived and designed the study. Lin G and Huang Y performed the literature search. Li R and Cao J performed the statistical analysis. Zhang Y and Huang Y wrote the manuscript. Dong M and Huang Z reviewed and edited the manuscript. Deng Y revised the manuscript. All authors read and approved the final version of the manuscript.

## Figures and Tables

**Table 1:** Basic information of the included studies.

Study	Country	Recruitment period	Median age	No. of patients	Histological type	TNM stage	Treatment type	Cut-off value (g/L)	Follow up (month)	Survival outcomes
Altiay et al. (15)	Turkey	2004.09-2005.09	61	78	NSCLC/SCLC	IIIA-IV	Chemotherapy, radiotherapy	3.80	NR	OS
Chen et al. (2)	China	2009.01-2010.12	62	182	NSCLC	I-III	Mixed	3.62	NR	OS*, PFS*
Fan et al. (31)	China	2011.01-2015.12	63.2	120	SCLC	NR	Mixed	4	NR	OS*
Gu et al. (16)	China	2012.07-2016.02	68	97	NSCLC	I-IV	NR	4	NR	OS*, PFS*
Huang et al. (17)	China	2006.01-2009.12	60	589	NSCLC	I-IIIA	Mixed	3.48	44	OS*, DFS*
Jiang et al. (18)	China	2008.01-2011.12	60	184	NSCLC	I-IIIA	Mixed	4	18.5	DFS*
Kim et al. (30)	Korea	2007.01-2011.12	66.3	854	NSCLC	IIIA-IV	Chemotherapy, radiotherapy	4.5	NR	OS*
Li et al. (20)	China	2001.01-2008.12	60.3	122	NSCLC	I-III	Mixed	4	36	OS*
Li et al. (19)	China	2005.02-2014.01	60.33	412	NSCLC	I-IV	Mixed	3.3	NR	OS
Ma et al. (21)	China	2004.03-2009.01	NR	405	NSCLC	I-IIIA	Mixed	4	NR	OS*
Qi and Fu (22)	China	2008.12-2013.12	58	539	NSCLC	I-IV	NR	3.98	NR	OS
Sheng et al. (23)	China	2006.10-2009.12	60	567	NSCLC	I-IIIB	Mixed	4	21	OS*, DFS*
Wang et al. (24)	China	2005.01-2013.12	60	134	NSCLC	I-IIIA	Mixed	4	22	OS*, DFS*
Zeng et al. (6)	China	2007.12-2012.10	61	856	NSCLC	I-IV	Mixed	3.7	NR	OS*
Zhang et al. (25)	China	2010.01-2010.12	60	118	NSCLC	IIIB-IV	Chemotherapy	4	NR	OS*
Zhu et al. (26)	China	2000.01-2011.05	56	275	NSCLC	NR	Mixed	5	20.7	OS*
Zhu (14)	China	2009.05-2014.08	57	74	SCLC	NR	Chemotherapy, radiotherapy	5.8	11.5	OS*, PFS*

Abbreviations: SCLC: small cell lung cancer; NSCLC: non-small cell lung cancer; NR: not reported; Mixed: treatment included surgery, chemotherapy and radiotherapy; OS: overall survival; DFS: disease-free survival; PFS: progression-free survival;

* Multivariate analysis.

**Table 2:** The Newcastle-Ottawa Scale (NOS) quality assessment of the eligible studies.

Study ID	Selection	Comparability	Exposure/Outcome	Total score
Altiay et al. (15)	•••	•	••	6
Chen et al. (2)	••••	•	•	7
Fan et al. (31)	••	••	•••	7
Gu et al. (16)	•••	•	••	6
Huang et al. (17)	••	•	•••	6
Jiang et al. (18)	••	•	•••	6
Kim et al. (30)	•••	••	••	7
Li et al. (20)	••	•	•••	6
Li et al. (19)	•••	•	••	6
Ma et al. (21)	••	•	•••	6
Qi et al. (22)	••	••	•••	7
Sheng et al. (23)	••	•	•••	6
Wang et al. (24)	••	•	•••	6
Zeng et al. (6)	•••	•	••	6
Zhang et al. (25)	••	•	•••	6
Zhu et al. (26)	••••	•	•	6
Zhu et al. (14)	•••	•	••	6
